# Social History Matters–The Impact of Illicit Drug Use on tPA Use and In-Hospital Mortality in Acute Ischemic Stroke

**DOI:** 10.9734/INDJ/2014/7708

**Published:** 2014-02-07

**Authors:** Matthew H. M. Marx, Karen C. Albright, Amir Shaban, Amelia K. Boehme, T. Mark Beasley, Sheryl Martin-Schild

**Affiliations:** 1 Department of Neurology, Tulane University Hospital, New Orleans, LA 70112, United States.; 2 Health Services and Outcomes Research Center for Outcome and Effectiveness Research and Education (COERE), USA.; 3 Center of Excellence in Comparative Effectiveness Research for Eliminating Disparities (CERED) Minority Health and Health Disparities Research Center (MHRC), USA.; 4 Department of Epidemiology, School of Public Health, University of Alabama at Birmingham, England.; 5 Department of Neurology, School of Medicine, University of Alabama at Birmingham, United States.; 6 Department of Biostatistics, School of Public Health, University of Alabama at Birmingham, England.

**Keywords:** Ischemic stroke, substance abuse, thrombolytic therapy, tissue plasminogen activator

## Abstract

**Aims:**

The objective of this descriptive study was to compare time to medical evaluation, intravenous tissue plasminogen activator (IV tPA) use, and short-term outcomes in illicit drug users compared to non-users presenting with acute ischemic stroke (AIS).

**Study Design:**

This is a retrospective study performed from our stroke registry using deidentified patient information.

**Place and Duration of Study:**

Tulane Medical Center Primary Stroke Center (PSC). Consecutive AIS patients presenting to our PSC from July 2008 to December of 2010 were identified from our prospectively collected stroke registry.

**Methodology:**

Patients were categorized as toxicology positive (TP) or toxicology negative (TN). We compared baseline characteristics, clinical presentation, tPA use, and short-term outcomes in TP and TN patients.

**Results:**

Two hundred and sixty-three patients met inclusion criteria (median age 63, 35.4% female, 66.5% Black). Nearly 40% of toxicology screens were positive. Stroke severity was similar with the median National Institute of Health Stroke Scale (NIHSS) of 6 in both groups; however, a higher proportion of TN patients were treated with IV tPA (32.1% vs. 21.2%). After adjustment for time from last seen normal to emergency department arrival (LSN-to-ED arrival), the odds of being treated with tPA for TP patients were similar to TN patients (OR 0.69, 95% CI 0.36–1.31, p=0.255). After adjustment for age, NIHSS, glucose, and tPA, the odds of in-hospital mortality in TP patients was 3 times that of TN patients (OR 3.17, 95% CI 1.07–9.43, p=0.038).

**Conclusion:**

We found that the disparities observed in tPA use were attenuated after adjustment for time from LSN-to-ED arrival, suggesting an area for future intervention. Additionally, we found that TP patients may be at higher risk for in-hospital mortality. Further study on the role of substance abuse in time to ED arrival, tPA use, and outcome in AIS patients is warranted.

## 1. INTRODUCTION

The 2011 National Survey on Drug Use and Health found that 6.3% of US adults, age 26 or older currently use illicit drugs [1]. Traditionally, screening for illicit drugs has been performed in younger stroke patients, as drug abuse may be the most common predisposing condition for stroke among patients under 35 years of age [2,3]. National survey data suggest that rates of illicit drug use among adults ages 50 to 59 have been increasing since 2002 [1]. This increase has been attributed to the aging the baby boom cohort in which increased drug use during their youth may be being continued into older age [1].

Little is known about the relationship between illicit drug use and time to emergency department arrival in the setting of acute ischemic stroke (AIS). Further, no study has investigated intravenous (IV) recombinant tissue plasminogen activator (tPA) use in illicit drug users compared to non-users. The objective of this descriptive study was to compare time to medical evaluation, tPA use, and short-term outcomes in illicit drug users compared to non-users presenting with AIS.

## 2. METHODOLOGY

### 2.1 Methods

Siemens Dimension Vista system with Flex reagent cartridges were used for urine drug screen, patients with the presence of one or more of the following illicit substances in their urine were categorized as toxicology positive (TP) the metabolites tested on urine toxicology screen are shown [as follows] when applicable: amphetamine [d-amphetamine, l-amphetamine, MDA, chloroamphetamine], barbiturates, benzodiazepines, cocaine [benzoylecgonine], methamphetamine, methadone [l-methadone, d-methadone], opiates, phencyclidine (PCP), or tetrahydrocannabinol (THC) [11-nor-9 carboxy-Δ9-THC, cannabinol]. The sensitivity and specificity for each compound screened are shown in the supplementary table found in the Appendix. The remaining patients were classified as toxicology negative (TN). Patients who did not have urine toxicology performed were excluded.

We compared baseline characteristics, time from last seen normal (LSN) to emergency department (ED) arrival, stroke severity (as measured by the National Institutes of Health Stroke Scale [NIHSS] score), treatment with intravenous (IV) recombinant tissue plasminogen activator (tPA), and short-term outcomes in TP and TN patients. Short-term neurologic deficits were estimated using the discharge NIHSS. Short-term functional outcomes were assessed using the modified Rankin scale (mRS) score. All NIHSS scores and mRS scores were performed by NIHSS and mRS certified physicians. The proportion of known and unknown LSN times and mean time from LSN to ED arrival were compared by illicit substance.

Categorical data were compared using Pearson Chi-squared (or Fisher exact test where appropriate). Continuous data were compared using the Student’s t-test (or Wilcoxon Rank Sum test where appropriate). Logistic regression was used to determine the odds of receiving IV tPA and the odds of in-hospital mortality. Crude and adjusted models were performed. All tests were performed at the α=0.05 level and were two-sided. We did not correct for multiple comparisons, as this was an exploratory study [4]. This cross-sectional study was approved by the institutional review board at the Tulane University.

### 2.2 Statistical Methods

Categorical data are presented as frequencies and were compared using Pearson Chi-squared or Fisher exact test where appropriate. Continuous data are presented as medians with ranges and were compared using Wilcoxon Rank Sum test. All tests were performed at the α=0.05 level and were two-sided. The retrospective chart review was approved by the institutional review board at the Tulane University (IRB protocol number 237137-3).

## 3. RESULTS

Five hundred and ninety-three patients were screened. Two hundred and sixty-three met inclusion criteria (median age 63, 35.4% female, 66.5% Black). A higher proportion of patients self-reporting a history of substance had a urine drug screen performed (25.3% vs. 8.7% p<0.001). Nearly 40% of toxicology screens performed were positive. Table 1 compares and contrasts TP and TN AIS patients. Table 2 shows the percent of patients with each positive result. There were no significant differences in the age, sex, race, or cardiovascular comorbidities in TP and TN patients (Table 1). A higher proportion of TP patients reported a history of substance abuse (p<0.001) and described themselves as current smokers (p=0.005). Stroke severity was similar with the median NIHSS 6 in both groups; however, a higher proportion of TN patients were treated with IV tPA (32.1% vs. 21.2%, p=0.053). A larger proportion of TN strokes were cardioembolic (26.6% vs. 18.3%, Table 1).

Comparison of known and unknown last seen normal times (LSN) in TP and TN AIS patients revealed that a nonsignificantly higher proportion of TP patients reported their LSN time as unknown (44.6% vs. 35.8%, p=0.209, Table 3). Among TP patients, a higher proportion of patients with evidence of recent opiate use reported their LSN time as unknown (21.5% vs. 5.9%, p=0.001). In contrast to opiate positive patients, recent THC use was associated with a lower proportion of patients with unknown LSN time (4.6% vs. 18.7%, p=0.006). Table 4 illustrates the mean time from LSN to ED arrival compared by illicit substance. Only cocaine positive patients demonstrated a significantly longer time to arrival (1772 vs. 815 minutes, p=0.024). The odds of being treated with IV tPA were lower for TP patients (crude odds ratio [OR] 0.57, 95% CI 0.32–1.01, p=0.055). After adjustment for time from LSN to ED, the odds of being treated with IV tPA for TP patients were attenuated (adjusted OR 0.69, 95% CI 0.36–1.31, p=0.255).

In the center where this study was conducted, a patient suffering an acute ischemia stroke can receive tPA if he or she presents within 4.5 hours of their last seen normal time. Of the TN patients (n=159), 34.0% (n=54) had a contraindication to tPA administration; of TP patients (n=104), 40.3% (n=42) had a contraindication to IV tPA administration. Thus, 88/159 (55.3%) TN and 78/104 (75.0%) TP had no contraindications to tPA use. Of those in the latter two groups, 52.3% (46/88) TN and 33.3% (26/78) of TP patients were given IV tPA. There was no difference in proportion of tPA treated patients who were treated within the first 3 hrs versus beyond 3 hrs based on toxicology screen positivity (p=0.777).

While short-term measures of neurologic deficits (NIHSS on discharge 2 vs. 3) and functional (median mRS 3 vs. 3) were similar in TP and TN patients, the proportion of TP patients who experienced in-hospital mortality was nonsignificantly higher (10.7% vs. 5.8%, Table 5). The odds of in-hospital mortality in TP patients were not significantly higher than that for TN patients (crude OR 1.95, 95% CI 0.78–4.89, p=0.153). The cause of death among TP patients was stroke-related in 73%. When compared with TN patients whose cause of death was stroke-related in 44%, TP was not significantly associated with stroke-related death (p=0.362). All other deaths in each group were due to cardiac causes. Given that AIS patient outcome has been associated with age, stroke severity, and glucose on admission, we examined the odds of in-hospital mortality in TP patients adjusting for these important confounders. The adjusted model found that the odds of in-hospital mortality in TP patients was 3 times that of TN patients (adjusted OR 3.19, 95% CI 1.08–9.42, p=0.036). This association remained when tPA was added to the model (adjusted OR 3.17, 95% CI 1.07–9.43, p=0.038).

## 4. DISCUSSION

Our study found that only 44% of AIS patients routinely received a urine drug screen, similar to a recent report where the proportion ranged from 37–43% [5]. This may be due to the traditional neurologic teaching to consider illicit substance use in cases of stroke in the young. With stroke incidence increasing with age and the assumption being that illicit drug use would decline in older age cohorts, one may conclude that checking for illicit substance abuse would be of lesser importance in the evaluation of older AIS patients. However, recent data suggest that substance abuse rates are rising in older adults, potentially reflecting the aging of the baby boom cohort in which drug use is continuing into older age [1]. This serves as a reminder to neurologists that they may need to reconsider their criteria for performing a urine drug screen [5].

Similar to a recent report where tPA was administered to none of the cocaine positive ischemic stroke patients compared to 11% of cocaine negative patients, our study found that a higher proportion of TP patients were not treated with tPA [5]. Contraindications to tPA administration include SBP > 185 or DBP > 110 mmHg, CT findings suggestive of Intracerebral hemorrhage (ICH) or subarachnoid hemorrhage (SAH), suspicion of SAH, seizure at onset of stroke, recent intracranial or spinal surgery, recent head trauma, stroke within 3 months, major surgery or trauma within previous three months, recent internal bleeding (less than 22 days), platelets < 100,000, heparin use within 48 hours with PTT > 40, INR > 1.7, known bleeding diathesis or major disorder associated with risk of bleeding, or history of intracranial hemorrhage, brain aneurysm, vascular malformation, or brain tumor [6]. Potential reasons for non-treatment may include meeting blood pressure exclusion criteria if on stimulants, or having altered level of consciousness which confounds the initial time to stroke diagnosis. However, this association was no longer present after adjustment for time from LSN to ED, suggesting that time to ED presentation may be confounding the association between illicit drug use and tPA administration. The reasons for delay in seeking care in substance abusers are likely multifactorial and a function of both individual and system barriers. Barriers to care for substance abusers have been well-described and grouped into economic limitations, geographic limitations, lack of integrated services, cultural differences (e.g., language barriers, cultural practices and beliefs), patient physician communication, stigmatization, and lack of trust, respect, and confidentiality [7,8].

Additionally, we found that the proportion of TP patients who experienced in-hospital mortality was higher than that of TN patients. After adjustment for age, NIHSS, glucose on admission, and tPA use, we found that the odds of in-hospital mortality in TP patients in our sample were 3 times that of TN patients. While this is in keeping with previous reports of higher mortality in drug users, it is also possible that the TP and TN groups were inherently different in measures not collected or not assessed by our study.

Our results should be interpreted with caution. Given that we acquired information on exposures and outcomes at the same time, we were unable to make causal inferences. Further, our relatively small sample size may not have allowed us to detect existing differences in groups. While our sample is representative of the population residing in the catchment area of our medical center, these findings may not be generalizable to the US population as a whole.

## 5. CONCLUSION

Despite several limitations, our study calls attention to the relevance of substance abuse in AIS patients of all ages. Combined with national survey data and a previous report, our study suggests that performing urine drug screening may be appropriate for stroke patients of all ages [5]. Additionally, it highlights disparities in TP and TN AIS treatment rates, offering one possible explanation for the problem—time to ED arrival, which may be drug-specific. Finally, our study suggests that TP patients may be at higher risk for in-hospital mortality. Additional study on the role of substance abuse in arrival times, tPA use, and outcome in AIS patients is warranted.

## Supplementary Material

01

## Figures and Tables

**Table 1:** Comparison of baseline characteristics of TP and TN AIS patients

Parameter	TN (n=159)	TP (n=104 )	P value
Demographics, No. (%)			
Age, years, median (range)	66 (23–90)	58 (23–90)	0.200
Female	52 (32.7%)	41 (39.4%)	0.265
Black Race	103 (64.8%)	70 (69.3%)	0.693
Past Medical History, No. (%)			
Prior stroke	61 (38.4%)	45 (43.3%)	0.428
HTN	119 (75.8%)	82 (80.4%)	0.386
DM	49 (31.0%)	30 (29.4%)	0.784
CAD	30 (18.9%)	18 (17.3%)	0.749
Hyperlipidemia/Dyslipidemia	69 (43.9%)	40 (39.6%)	0.490
Social History, No. (%)			
History of substance abuse	21 (13.3%)	45 (43.7%)	<0.001
Active Smoker	55 (35.3%)	53 (53.0%)	0.005
Stroke Characteristics, No. (%)			
Admission NIHSS, median (range)	6 (0–31)	6 (0–31)	<0.001
Admission glucose, median (range)	121 (70–663)	115 (46–625)	0.361
IV tPA	51 (32.1%)	22 (21.2%)	0.053
TOAST			
Cardioembolic	42 (26.6%)	19 (18.3%)	0.004
Large Vessel	47 (29.7%)	22 (21.2%)	
Small Vessel	32 (20.3%)	22 (21.2%)	
Other	5 (3.2%)	17 (16.3%)	
Crypto (unknown)	28 (17.7%)	20 (19.2%)	

Table 1 Shows demographics, patient past medical history, social history, stroke characteristics, and TOAST scale; these parameters are relevant to the patients’ presentations. NIHSS = National Institute of Health Stroke Scale, IV tPA = Intravenous tissue plasminogen activator, TOAST = Trial of Org 10172 in Acute Ischemic Stroke Treatment

**Table 2:** The number of patients who tested positive for each drug

Drug of abuse	Number of patients positive (percent of total patients screened)	Number of patients positive (percent of patients with positive toxicology screen)
Benzodiazepines	30 (11.4%)	30 (24.8%)
Cocaine	28 (10.6%)	28 (23.1%)
Opiate	26 (9.8%)	26 (21.5%)
THC	38 (14.1%)	38 (31.4%)
Barbiturate	7 (2.7%)	7 (6.7%)
MDMA	2 (0.7%)	2 (1.9%)

Table 2 Shows the number of patients who tested positive for each drug from the total number of patients screened (n = 263) and the number of patients who tested positive for each drug from all patients who had any positive toxicology result (n=104).

**Table 3:** The last seen normal (LSN) for patients presenting to the emergency department

Illicit Substance Toxicology Screen	Unknown LSN (n=65)	Known LSN (n=187)	P value
Any Tox Positive	29 (44.6%)	67 (35.8%)	0.209
Benzodiazepines	9 (13.8%)	16 (8.6%)	0.219
Cocaine	8 (12.3%)	20 (10.7%)	0.722
Opiates	14 (21.5%)	11 (5.9%)	0.001
THC	3 (4.6%)	35 (18.7%)	0.006

Table 3 Shows the last seen normal (LSN) for patients with acute ischemic stroke presenting to the emergency department. Unknown LSN time excludes patients from IV tPA administration, as it is unknown if they are within the treatment window. Other illicit substances were not analyzed due to small numbers of patients.

**Table 4:** Median last seen normal (LSN) to emergency department (ED) arrival time for each drug

Toxicology Screen Results	LSN to ED arrival (minutes), median (range)	P value
Any Tox positive (n=75)	508 (39–17351)	0.2095
Tox negative (n=128)	252 (20–9280)	
Benzo positive (n=21)	52 (35–1510)	<0.0001
Benzo negative (n=182)	345 (27–17351)	
Cocaine positive (n=20)	801 (50–17351)	0.0084
Cocaine negative (n=183)	250 (18–9280)	
Opiate positive (n=13)	722 (43–1305)	0.5435
Opiate negative (n=190)	264 (18–17351)	
THC positive (n=35)	626 (18–7293)	0.0616
THC negative (n=168)	250 (15–17351)	

Table 4 Shows the breakdown of patients with whom the last seen normal (LSN) to emergency department (ED) arrival time was known and the results of the toxicology screen in these patients. Benzo = benzodiazepines. Other illicit substances were not analyzed due to small numbers of patients.

**Table 5:** Comparison of outcomes of TP and TN AIS patients

Outcome Parameters	TN (n=159)	TP(n=104)	p value
Length of Stay, days, median (range)	6 (1–95)	6 (1–34)	<0.001
Discharge NIHSS, median (range)	2 (0–42)	3 (0–42)	<0.001
Discharge mRS, median (range)	3 (0–6)	3 (0–6)	<0.001
Discharge mRS 0–1	44 (28.2%)	25 (24.3%)	0.483
Discharge mRS 0–2	61 (39.1%)	41 (39.8%)	0.910
Discharge disposition			
Home	75 (47.5%)	53 (51.0%)	0.431
Inpatient Rehab	52 (32.9%)	27 (26.0%)	
LTAC	10 (6.3%)	3 (2.9%)	
Skilled Nursing	7 (4.4%)	7 (6.7%)	
Hospice	4 (2.5%)	3 (2.9%)	
Other	1 (0.6%)	0	
Expired	9 (5.8%)	11 (10.7%)	
In-hospital Mortality	9 (5.8%)	11 (10.7%)	0.147

Table 5 Shows the outcomes for all patients who were given a urine toxicology in the emergency department. tn = toxicology negative, tp = toxicology positive, NIHSS = National Institute of Health Stroke Scale, mRS = modified Rankin Scale, ltac = long term acute care.

## References

[1] (2012). Substance abuse and mental health services administration, results from the 2011 national survey on drug use and health: Summary of national findings.

[2] Esse K, Fossati-Bellani M, Traylor A, Martin-Schild S (2012). Epidemic of illicit drug use, mechanisms of action/addiction and stroke as a health hazard. Brain Behav.

[3] De los Rios F, Kleindorfer DO, Khoury J, Broderick JP, Moomaw CJ, Adeoye O (2012). Trends in substance abuse preceding stroke among young adults: A population-based study. Stroke.

[4] Bender R, Lange S (2001). Adjusting for multiple testing--when and how?. J Clin Epidemiol.

[5] Silver B, Miller D, Jankowski M, Murshed N, Garcia P, Penstone P (2013). Urine toxicology screening in an urban stroke and tia population. Neurology.

[6] Cocho D, Belvis R, Marti-Fabregras J, Molina-Porcel L, Diaz-Manera J, Aleu A (2005). Reasons for exclusion from thrombolytic therapy following acute ischemic stroke. Neurology.

[7] (2002). Manual for primary care providers: Effectively caring for active substance users.

[8] Monks R, Topping A, Newell R (2013). The dissonant care management of illicit drug users in medical wards, the views of nurses and patients: A grounded theory study. J Adv Nurs.

