# Supplementary material for: Social History Matters–The Impact of Illicit Drug Use on tPA Use and In-Hospital Mortality in Acute Ischemic Stroke
Source: Int Neuropsychiatr Dis J. Author manuscript; Available in PMC 2014 Nov 26. (PMC4245033; doi:10.9734/INDJ/2014/7708)
Supplement: 01 [file NIHMS570598-supplement-01.pdf]

APPENDIX

Supplementary Table

	Sensitivity	Specificity	Sensitivity for detection (ng/mL)
Amphetamine	95%	100%	125
Barbiturates	100%	90%	20
Benzodiazepines	100%	100%	30
Cocaine	100%	100%	35
EXTC-MDMA	100%	95%	75
Methamphetamine	96%	100%	107
Opiates	87%	100%	50
PCP	100%	100%	5
THC	100%	96%	15

Supplementary Table shows the sensitivity, specificity, and sensitivity for detection for each drug using our toxicology methodology.

© 2014 Marx et al.; This is an Open Access article distributed under the terms of the Creative Commons Attribution License (http://creativecommons.org/licenses/by/3.0), which permits unrestricted use, distribution, and reproduction in any medium, provided the original work is properly cited.

Peer-review history:
The peer review history for this paper can be accessed here:
http://www.sciencedomain.org/review-history.php?iid=415&id=29&aid=3573
